# Unveiling sex-based differences in developing propionic acid-induced features in mice as a rodent model of ASD

**DOI:** 10.7717/peerj.15488

**Published:** 2023-06-13

**Authors:** Nasreen Kamalmaz, Abir Ben Bacha, Mona Alonazi, Gadah Albasher, Arwa Ishaq A. Khayyat, Afaf El-Ansary

**Affiliations:** 1 Biochemistry Department, Science College, King Saud University, Riyadh, Saudi Arabia; 2 Zoology Department, Science College, King Saud University, Riyadh, Saudi Arabia; 3 Central Research Laboratory, King Saud University, Riyadh, Saudi Arabia

**Keywords:** Autism, Sex difference, Propionic acid, Behavior

## Abstract

**Background:**

Males are more likely to develop autism as a neurodevelopmental disorder than females are, although the mechanisms underlying male vulnerability are not fully understood. Therefore, studying the role of autism etiologies considering sex differences in the propionic acid (PPA) rodent model of autism would build greater understanding of how females are protected from autism spectrum disorder, which may be used as a treatment strategy for males with autism.

**Objectives:**

This study aimed to investigate the sex differences in oxidative stress, glutamate excitotoxicity, neuroinflammation, and gut microbiota impairment as etiological mechanisms for many neurological diseases, with specific reference to autism.

**Method:**

Forty albino mice were divided into four groups of 10 animals each with two control and two treated groups of both sexes received only phosphate-buffered saline or a neurotoxic dose of PPA (250 mg/kg body weight) for 3 days, respectively. Biochemical markers of energy metabolism, oxidative stress, neuroinflammation, and excitotoxicity were measured in mouse brain homogenates, whereas the presence of pathogenic bacteria was assessed in mouse stool samples. Furthermore, the repetitive behavior, cognitive ability, and physical-neural coordination of the animals were examined.

**Results:**

Collectively, selected variables related to oxidative stress, glutamate excitotoxicity, neuroinflammation, and gut bacteria were impaired concomitantly with altered behavior in PPA-induced rodent model, with males being more susceptible than females.

**Conclusion:**

This study explains the role of sex in the higher vulnerability of males to develop autistic biochemical and behavioral features compared with females. Female sex hormones and the higher detoxification capacity and higher glycolytic flux in females serve as neuroprotective contributors in a rodent model of autism.

## Introduction

Autism spectrum disorder (ASD) is a complex neurodevelopmental disorder that impairs brain development and typically manifests within the first three years of life (Estes et al., 2015). The reported incidence of ASD has risen dramatically over the last decade to one in 54 children and is more prevalent in boys at a 4:1 ratio (Maenner et al., 2020). ASD is distinguished by impaired social communication and repetitive and restrictive behavior (Baio et al., 2018). Treatments to cure the primary clinical symptoms of ASD remain unavailable. Although the exact etiological mechanisms of ASD are difficult to define, it is agreed that the occurrence of several or all the numerous factors comprising genetic, immune, nutritional, and environmental factors could contribute to the etiology of ASD (Abuaish et al., 2021a; Abuaish et al., 2021b; Zurita et al., 2020; Sealey et al., 2016; Kreiser & White, 2014).

Antibiotic overuse causes dysbiosis and inflammation that contributes to the pathophysiology of gastrointestinal diseases and autistic traits (Navarro, Liu & Rhoads, 2016). To create ASD-like animal models, propionic acid (PPA), a gut metabolic end product, can be administered and causes oxidative stress, mitochondrial dysfunction, neuroinflammation, and abnormal neurobehaviors as well as repetitive and poor social interactions in the rodent model (Abuaish et al., 2021a; Choi et al., 2018; Nankova et al., 2014; El-Ansary, Bacha & Kotb, 2012; MacFabe et al., 2011; Shultz et al., 2008).

The gut microbiota is a mutually beneficial bacterial habitat that is required for host survival and has a significant impact on the brain and behavior (Fattorusso et al., 2019; Ristori et al., 2019; Coretti et al., 2018) *via* the bidirectional gut-brain axis (GBA) that connects the gut to the central nervous system (CNS) (Martin et al., 2018; Borrelli et al., 2016). The GBA is reported to play an important role in social behavior management and in regulating myelin in the prefrontal cortex, a brain area that controls cognitive behavior (Bastiaanssen & Cryan, 2021; Roman et al., 2018). Males and females have sexually dimorphic patterns in energy and nutritional requirements throughout their lives, and therefore, gender differences in the gut microbiome-brain axis may be an important biological factor (Bolnick et al., 2014; Schnorr et al., 2014; Kaplan et al., 2000).

Oxidative stress occurs is caused by a lack of balance between the generation of reactive oxygen/nitrogen species (ROS) and the blocking of an damaging impacts through the use of antioxidative systems (Pangrazzi, Balasco & Bozzi, 2020). Oxidative stress is strongly considered to play a significant role in ASD pathophysiology (Bjørklund et al., 2020; Nadeem et al., 2019; Wang et al., 2018), and oxidative stress is critical to neuroinflammatory response, which has always been considered as a pathogenic factor of ASD (Herbert, 2011). Rose et al. (2017) found that children with autism are more vulnerable to oxidative stress because of an imbalance in glutathione (GSH) levels inside or outside of cells and a reduced GSH storage capacity compared with that in healthy siblings (Rose et al., 2017; Hu et al., 2020; Bjørklund et al., 2016). Estradiol is a steroidal hormone that plays a critical role in mitochondrial function and neuronal signaling and has been lately discovered to act as a neuroprotectant only under specific conditions (Engler-Chiurazzi, Singh & Simpkins, 2016; Bruce-Keller et al., 2000; Lebesgue et al., 2009). A key protective action of estradiol is the ability to scavenge free radicals (Prokai-Tatrai et al., 2009; Simpkins et al., 2010), and this antioxidant activity has been linked to a sex difference in oxidative stress. Oxidative damage to DNA and lipid was found to be higher in the liver mitochondria and synapsis of male rats than that in females (Borrás et al., 2003).

Glutamate (Glu) is the main excitatory neurotransmitters in the CNS, where it is involved in signal transmission and has a role in memory, learning, and synaptic plasticity (Parkin et al., 2018). However, high levels of extracellular Glu are linked to neuronal death, and Glu concentration is controlled by astrocytic Glu transporters, which remove Glu from the synapse after impulse transmission (Jack et al., 2018; Jia et al., 2015). By contrast, the main inhibitory neurotransmitter, gamma-aminobutyric acid (GABA), causes hyperpolarization of the postsynaptic cell membrane, dampening the action potential (El-Ansary et al., 2017; Ford, Nibbs & Crewther, 2017). Increased levels of Glu are found with decreased levels of GABA and glutamine (Gln) and the Glu/GABA and Glu/Gln ratios in the plasma and brains of children with autism (El-Ansary et al., 2017; Tu, Chen & He, 2012). Investigations on sex differences in excitotoxicity have been performed by studying the neuro-protective effects of estrogen and prolactin as female sex hormones against hippocampal neurodegeneration (Morales et al., 2014; Tejadilla, Cerbón & Morales, 2010). Prolactin treatment in ovariectomized females has been indicated to minimize hippocampus neurodegeneration (Morales et al., 2014; Tejadilla, Cerbón & Morales, 2010). Particularly during phases of dynamic hormonal change, such as puberty, testosterone may have a key impact in how ASD manifests and multiple studies have suggested that estrogens are potent antioxidants that can prevent oxidative damage in cell culture systems and may inhibit Glu-induced excitotoxicity (Silva et al., 1999; Muscatello et al., 2022).

Neuroinflammation is a chronic glial reaction occurring in the CNS that can cause brain damage by enhancing proinflammatory cytokine release and abnormal neuronal growth (Goines & Ashwood, 2013; Qasem, Al-Ayadhi & El-Ansary, 2016). Analysis of interleukins (IL) has demonstrated that enhanced inflammatory activity is present in children with autism. Levels of proinflammatory cytokines were found to be higher in the plasma of children with ASD in comparison with those in age-matched healthy children and children with other developmental disabilities. This phenomenon is linked to behavioral impairments, implying that dysfunctional immune responses may affect core behaviors in ASD (Ashwood et al., 2011).

Various factors may cause brain-based sex differences in ASD, for instance, endocrine and genetic mechanisms that “masculinize” and “feminize” the brain at early development, puberty, and other life-cycle hormone transition periods. Moreover, the brain’s arousal system and the stress axis may interact with sex-related biology to cause different neurodevelopmental patterns in females and males with ASD.

This information prompted us to investigate the gender differences in a number of biochemical and behavioral factors, including gut microbiota, oxidative stress, Glu excitotoxicity, and neuroinflammation in an ASD mouse model, to help understand why males are more likely than females to develop autistic traits and the protective mechanisms present in females. The current mouse model, differs from our previously used rat models (Abuaish et al., 2021a; Abuaish et al., 2021b; El-Ansary, Bacha & Kotb, 2012) and has been crucial in advancing our understanding of the behavioral impairments and physiology linked to sex differences in ASD and has enabled us to prepare the foundations of future research and new therapeutic approaches. We hypothesized that mice may make favorable models because they exhibit behavioral patterns and differences in most brain areas that are comparable with those reported in people with autism (Kazdoba et al., 2016).

As a preliminary investigation in mice with an ASD mouse model, sex differences were examined in relation to behavioral abnormalities in both sexes parallel with levels of oxidative stress, Glu signaling, neuroinflammation, and alterations to gut bacteria as ascertained with etiological mechanisms in ASD (Nadeem et al., 2019; Wang et al., 2018; Ford, Nibbs & Crewther, 2017; Aabed et al., 2019).

## Material and Methods

### Animal experiments

A total of 40 young (2–3-week-old) male and female albino mice (*Mus musculus*) weighing 13–15 and 11–13 g, respectively, were kept in cage (40 × 5 × 20 cm^3^) at a controlled temperature (21 ± 1 °C) under standard laboratory conditions (humidity 37% and light for 12 h) with ad libitum access to standard laboratory animal feed pallets and water. Mice were obtained from the animal house in the Zoology Department in Science College, King Saud University, Riyadh, KSA. The experimental procedure was preapproved by the ethics committee for animal research of King Saud University, Riyadh (ethics reference number: KSU-SE-19-92).

**Figure 1:**
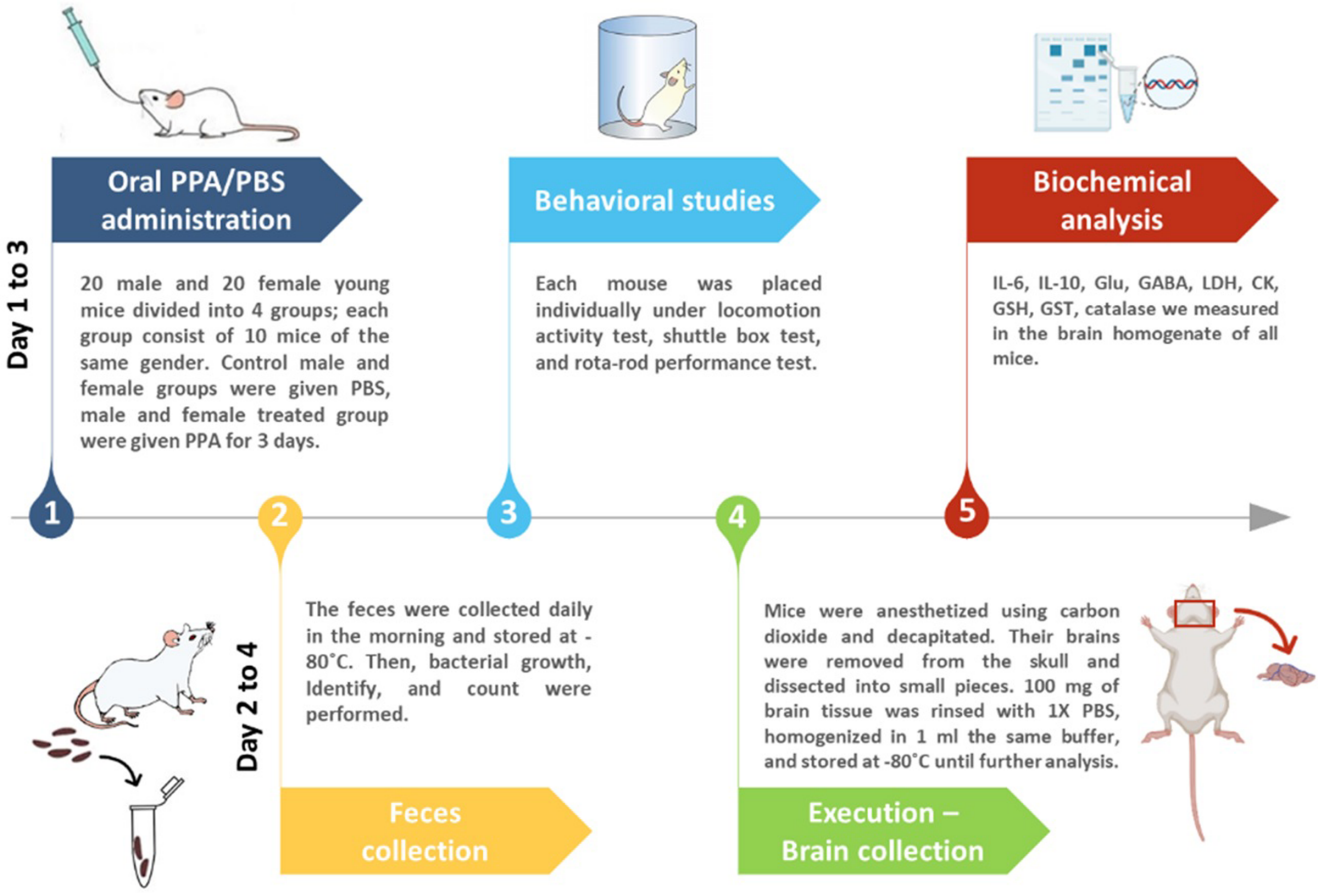
Diagrammatic scheme of the animal experiments.

Mice were divided into four groups of 10 animals each with two control and two treated groups of both sexes receiving phosphate-buffered saline (PBS) or an oral neurotoxic dose (250 mg/kg body weight) of PPA for 3 days, respectively (Fig. 1).

At the end of the experiment, mice were anesthetized using carbon dioxide and decapitated. Then, the whole brains of all groups were dissected into small pieces after being removed from the skull. Brain tissue (0.1 g) was rinsed with PBS and homogenized in one mL of PBS and kept at −20 °C.

### Biochemical analyses

Catalase and glutathione-S-transferase (GST) activities were assessed according to the methods of  Maehly & Chance (1954) and Habig, Pabst & Jakoby (1974), respectively. Lactate dehydrogenase (LDH) and creatine kinase (CK) total activities were assessed using diagnostic kits made by the United Diagnostics Industry, Dammam, KSA (Szasz, Gruber & Bernt, 1976; Amador, Dorfman & Wacker, 1963). The level of GSH in the brain samples was measured according to the protocol described by Beutler, Duron & Kelly (1963) using a UV-visible spectrophotometer (Ultraspec 2100 Pro, Amersham Biosciences, Amersham, United Kingdom).

IL-6 (Catalog No MBS824703, MyBioSource, San Diego, CA, USA), IL-10 (Catalog No MBS704754, MyBioSource), Glu (Catalog No MBS2601720, MyBio-Source), and GABA (Catalog No MBS723819, MyBioSource) were investigated using enzyme-linked immunosorbent assay kits following the manufacturers’ instructions. All measurements were performed at least in duplicate, and the mean of different readings was calculated. Quality control assays were performed to evaluate the experimental reproducibility through the inter- and intra-assay coefficients of variability (%CV).

### Microbial analysis

Sample collection: The fecal contents of all groups were collected on a daily basis in the morning in sterile tubes and immediately stored at −80 °C. To isolate and determine the bacterial etiologies of an infection as quickly and precisely as possible, bacteria were cultured utilizing the best artificial media and incubation conditions.

#### Techniques of bacterial culture

Fecal specimens were homogenized for 5 s in a vortex mixer (Intalb, Santa Clara, CA, USA) before being centrifuged for 3 min at 4,000 rpm at −4 °C (AllegraTM 21R, Beckman, Brea, CA, USA). Following centrifugation, tenfold serial dilutions of the fecal suspensions were performed by adding one mL of the supernatant to nine mL of PBS (dilution 1). The process was repeated until dilution 4 was reached. Then, 0.1 mL of each of the prepared dilutions was smeared on the surface of five different culture media: nutrient agar (NA) for the isolation of *Staphylococcus* and/or *Bacilli* (gram-positive or -negative rod), Macconkey agar (MCA) for distinguishing *Enterobacteriacea* (gram-negative rod, lactose fermenters), Mueller Hinton agar (MHA) to identify *Moraxella* spp. (gram-negative), blood agar for distinguishing gram-positive/negative rod and cocci, and sulfite polymyxin sulfadiazine (SPS) agar to distinguish *Clostridium botulinum*. NA, MCA, MHA, and blood agar plates were incubated at 37 °C for 24 h under aerobic conditions, whereas SPS agar plates were incubated at 35 ± 2 °C for 24–48 h anaerobically (Li et al., 2015).

#### Quantification

Data from the culture-based methods were quantified based on a 1–4 scale defined as colony-forming unit (CFU), a measure of viable bacterial or fungal numbers. Unlike direct microscopic counts where all cells, dead and living, were counted, the CFU only measures viable cells. The number of CFUs on the plates was counted per dilution. Numbers between 30 and 300 were used to estimate the cultural count.

#### Identification

Every well-isolated colony, regardless of appearance, was picked in succession. If more than one colony type or more than one morphotype was observed, an attempt was made to isolate and characterize each of these. Each isolate was characterized according to the Holdeman, Moore & Cato (1977) procedure. Colonies were spread on the slide. Smears were heat-fixed and gently gram stained using 70% alcohol, crystal violet, safranin, and iodine. Slides were examined under a microscope using an oil immersion lens to identify these strains as gram-positive (violet color) or gram-negative (red color) and to whether they were cocci or bacilli.

### Behavioral study

#### Locomotion activity test

The locomotor activity test was performed in a square-shaped wooden arena (30 × 80 × 80 cm) with 64 squares painted on the arena floor (Ugo Basile, Italy). Several parameters were measured, including the number of squares crossed, washing, wall rearing, duration of locomotion, and immobility. Each mouse was placed in the experimental arena for 5 min under visual observations (Ajarem & Ahmad, 1998).

#### Shuttle box test

A shuttle box (Ugo Basile, Italy) was used to measure mouse avoidance reactions, which indicate mouse learning and memorizing abilities. The rectangular shuttle box was divided equally by a stainless-steel partition into two chambers with a gate providing access between the chambers. Each mouse was given 2 min with no stimulus to adapt to and familiarize itself with the shuttle box before beginning the trial sessions. A light bulb (21 W) and a buzzer (670 Hz and 70 dB) were turned on for 6 s consecutively as a conditioned stimulus (CS). After 5 s of CS, an unconditioned stimulus (US) that is electric scrambler shock (1 mA) was applied to the metallic grid floor for 4 s. The floor was a two-way procedure, so the shock (US) was delivered on either side of the metallic grid floor following the light and sound stimuli (CS). If the mouse avoided the US by escaping into the other chamber within 5 s after the CS, the microprocessor recorder unit of the shuttle box recorded an avoidance response. Each mouse underwent 30 trials with an intertrial interval of 15 s. The total number of avoidance responses and the total time taken to enter the other chamber to avoid the shock (latency of avoidance response or escape latency in seconds) were measured per mouse (Abu-Taweel, Ajarem & Ahmad, 2014).

#### Rotarod performance test

Here, a rotarod device (Ugo Basile, Italy) was used, with the mouse placed on a horizontally oriented rod that was mechanically rotating at 15 rpm. The rod is placed with at a low enough distance above the cage floor to avoid causing harm to the animal when they fall but high enough to encourage the animal to hold on and avoid falling. The amount of time an animal spends on this rotating rod is a measure of their physical ability, motor activity, balance, and coordination capability between the nervous and muscular systems (Maodaa et al., 2016).

### Statistical analysis

Data were presented as the average ± standard deviation (SD). Using Dunnett’s test for multiple comparisons, one-way analysis of variance tests was performed for all statistical comparisons between the studied groups. The Statistical Package for the Social Sciences (Chicago, IL, USA) was used to calculate the statistical analyses, and *p* values less than 0.05 were considered significant.

## Results and Discussion

Autism, as a neurodevelopmental condition, exhibits neuroinflammation in several brain areas. Activated astrocytes and microglia and elevated levels of proinflammatory cytokines demonstrate clear evidence of neuroinflammation in rodent models and individuals with autism (Aabed et al., 2019; Nadeem et al., 2022; Kern et al., 2015; El-Ansary & Al-Ayadhi, 2012).

Males and females show diverse functional correlations of neuroimmune signaling, which could be attributed to sexual dimorphism in cytokine activation in the brain (Hudson, Jacobson-Pick & Anisman, 2014). Generally, the female immune system includes numerous protective mechanisms that promote a stronger neuroimmune response when compared with that of the male immune system (Fransen et al., 2017).

**Figure 2:**
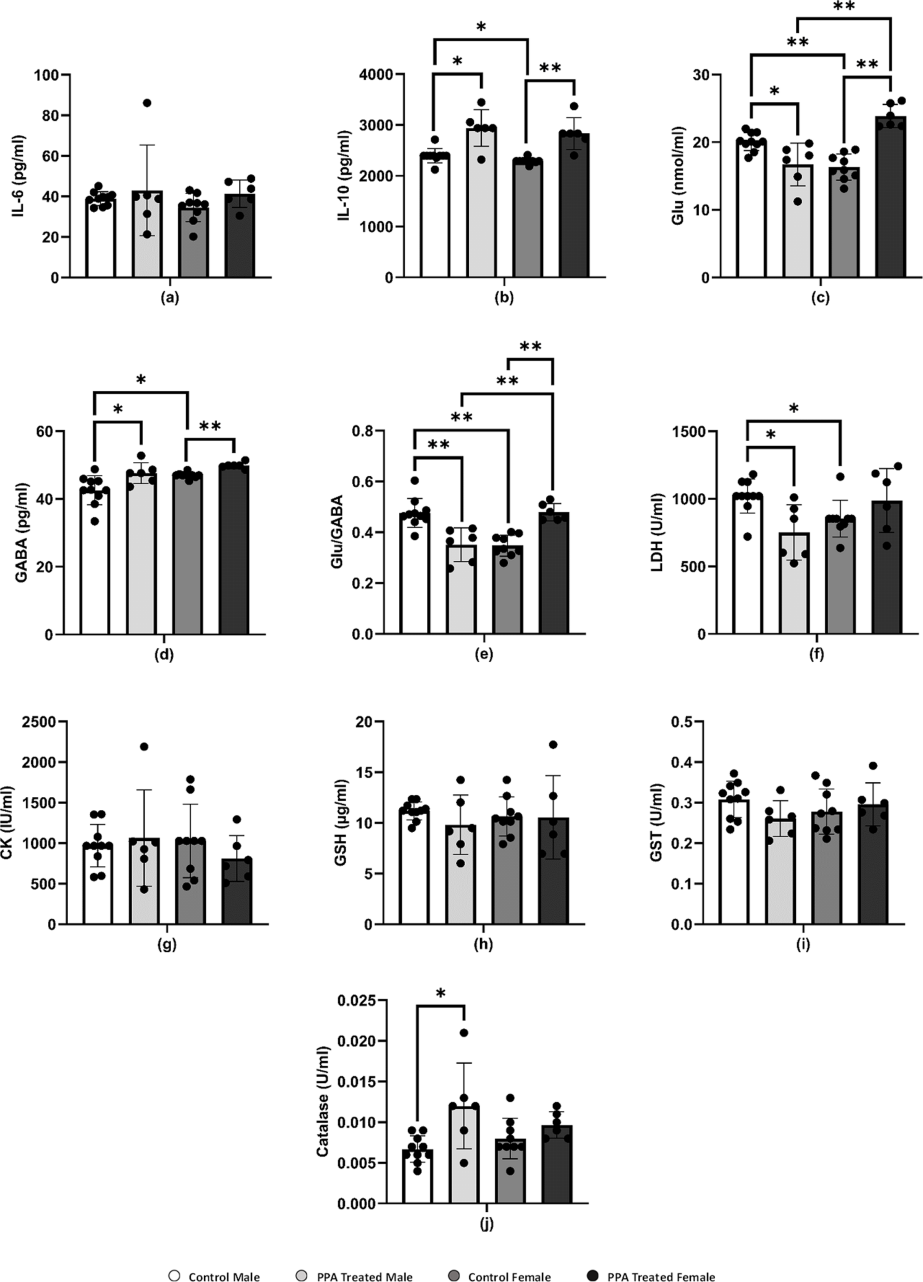
Comparing the levels of biochemical parameters in PPA-treated male and female mice to the comparable control groups. One asterisk (*), two asterisks (**), and three asterisks (***) show statistically significant differences at *P* < 0.05, *P* < 0.01 and *P* < 0.001, respectively from the control group by *t*-test.

In this study, females and males showed nonsignificant immune responses to PPA-induced neurotoxicity. Both sexes of PPA-treated mice showed approximately comparable IL-6 levels to those in the respective control groups (Table 1 and Fig. 2A). However, in contrast to the expected result, the level of IL-10 was significantly increased in both male (2,939.40 ± 361.78 pg/ml) and female (2,831.70 ± 311.96 pg/ml) mice (Table 1 and Fig. 2B). Studies by Villa et al. (2015) and Abuaish et al. (2021a) suggested that increased IL-10 levels in PPA-treated female rats act as protective mechanisms *via* the female sex hormone, estrogen, to activate the IL-10 promoter to support a significantly higher increase in IL-10 levels in females (*P* < 0.001) compared with a relatively lower elevation in males (*P* < 0.017). This could explain the resistance of female mice to developing biochemical and behavioral autistic characteristics after PPA exposure (Abuaish et al., 2021a). In addition, the anti-inflammatory effects of the physiological level of Glu *via* dendritic cell stimulation of T-reg cells to produce IL-10 could support the relationship between neurotransmission and immune response (Van Sadelhoff et al., 2019). The significant increase in IL-10 levels in PPA-treated male mice did not agree with the recent study by Abuaish et al. (2021a), which demonstrated lower IL-10 levels in PPA-treated males. This could be attributed to the differences in rats and mice being used as rodent models of ASD, especially regarding social behavior and rewarding.

**Table 1:** Mean ± S.D of all the measured parameters in brain homogenate of PPA treated male and female mice groups compared to control group. Only *P* values ≤0.05 were considered significant.

**Parameters**	**Groups**	**N**	**Min.**	**Max.**	**Mean ± S.D.**	**P value** ^a^	***P* value** ^b^	
**IL-** **6** **(pg/ml)**	Control Male	10	34.25	45.13	38.90 ± 3.41	0.786	0.688
	PPA Treated Male	6	21.27	86.11	42.99 ± 22.36			
	Control Female	9	20.21	43.03	34.50 ± 6.98	0.084		
	PPA Treated Female	6	30.49	48.76	41.29 ± 6.74			
**IL-10 (pg/ml)**	Control Male	10	2121.20	2712.10	2395.60 ± 140.33	0.017	0.593
	PPA Treated Male	6	2318.20	3444.40	2939.40 ± 361.78			
	Control Female	9	2186.90	2409.10	2287.90 ± 56.23	0.001		
	PPA Treated Female	6	2399.00	3368.80	2831.70 ± 311.96			
**Glu (nmol/ml)**	Control Male	10	17.68	22.00	20.09 ± 1.33	0.046	0.001
	PPA Treated Male	6	11.24	19.81	16.70 ± 3.15			
	Control Female	9	13.14	18.88	16.30 ± 1.93	0.001		
	PPA Treated Female	6	22.37	26.13	23.87 ± 1.71			
**GABA** **(pg/ml)**	Control Male	10	33.43	48.78	42.59 ± 4.30	0.026	0.123
	PPA Treated Male	6	43.63	52.77	47.62 ± 3.11			
	Control Female	9	45.34	48.50	46.95 ± 0.82	0.001		
	PPA Treated Female	6	48.62	51.41	49.84 ± 0.90			
**LDH** **(U/ml)**	Control Male	10	720.73	1182.17	1020.42 ± 126.37	0.021	0.094
	PPA Treated Male	6	522.97	1010.77	751.12 ± 204.27			
	Control Female	9	637.23	1164.59	852.58 ± 136.27	0.246		
	PPA Treated Female	6	652.61	1241.49	987.34 ± 236.88			
**CK** **(IU/ml)**	Control Male	10	582.29	1357.95	968.29 ± 261.51	0.660	0.337
	PPA Treated Male	6	430.68	2190.74	1063.88 ± 593.49			
	Control Female	9	465.83	1786.43	1027.49 ± 453.95	0.321		
	PPA Treated Female	6	509.78	1292.03	811.55 ± 283.89			
**GSH** **(µg/ml)**	Control Male	10	9.49	12.34	11.19 ± 0.88	0.307	0.873
	PPA Treated Male	6	6.01	14.24	9.81 ± 2.93			
	Control Female	9	7.91	14.24	10.64 ± 1.92	0.954		
	PPA Treated Female	6	6.96	17.72	10.55 ± 4.11			
**GST** **(U/ml)**	Control Male	10	0.23	0.37	0.31 ± 0.04	0.060	0.234
	PPA Treated Male	6	0.21	0.33	0.26 ± 0.04			
	Control Female	9	0.21	0.37	0.28 ± 0.06	0.545		
	PPA Treated Female	6	0.23	0.39	0.30 ± 0.05			
**Catalase (U/ml)**	Control Male	10	0.00	0.01	0.007 ± 0.002	0.012	0.225
	PPA Treated Male	6	0.01	0.02	0.012 ± 0.005			
	Control Female	9	0.00	0.01	0.008 ± 0.003	0.205		
	PPA Treated Female	6	0.01	0.01	0.010 ± 0.001			

**Notes.**

a *P* value between control and corresponding PPA traded group.

b *P* value between PPA treated groups.

Several neuropsychiatric conditions, including ASD, have been recently connected to glutamatergic system dysfunction. Changes in the gut microbiota and Glu metabolism in patients with ASD provide evidence supporting the Glu-centered hypotheses of ASD as demonstrated in postmortem and patient samples and studies, which demonstrated abnormalities in glutamatergic gene expression and metabolic pathways (Hollestein et al., 2023; Montanari et al., 2022). Over excitation of the Glu and glutamatergic receptors N-methyl-D-aspartate (NMDA) and α-amino-3-hydroxy-5-methyl-4-isoxazolepropionic (AMPA) acid activates enzymes that damage membrane permeability, electrochemical gradients, and cellular structure (Essa et al., 2013). Accordingly, several studies showed that people with ASD have lower levels of Glu metabolites in their basal ganglia and the anterior cingulate cortex and higher levels of Glu in their plasma (Cai et al., 2016; Tebartz Van Elst et al., 2014). Gln and Glu are related to each other in terms of structure and their immunomodulatory capabilities, and they are both necessary for intestinal growth and function. Lower plasma levels of Gln with concomitant higher levels of Glu have been reported in patients with ASD compared with those in healthy individuals. In this study, this was apparent with increased levels of Glu in PPA-treated female mice (Table 1 and Fig. 2C), which is not in good agreement with Al-Suwailem, Abdi & El-Ansary (2018), who reported a significantly lower level of Glu in PPA-treated female rats compared with that in males. Despite this increase, this result does not contradict the lower vulnerability of female rats to develop biochemical autistic features as Glu-induced toxic effects only occur at much higher neuroexcitatory levels. This explanation can be supported by considering that control female rats had significantly less Glu than that in control males, which may be related to the significantly higher gene expression of Glu transporters in females that can prevent microglial Glu-induced neuronal death with PPA treatment (Liang et al., 2008; Wickens, Bangasser & Briand, 2018).

GABA is the most abundant inhibitory neurotransmitter in the brain (Marshall, 2008). γ-Aminobutyric acid type A (GABA_A_) receptors, which are targeted by female sex hormones, are responsible for most inhibitory GABAergic effects in the brain (Bäckström et al., 2014). While estrogen reduces GABA inhibitory input, progesterone, and its neuroactive metabolites, allopregnanolone and pregnanolone, appear to increase the GABAergic inhibitory transmission *via* GABA_A_ receptor activation (Deligiannidis et al., 2013). Al-Suwailem, Abdi & El-Ansary (2018) showed that female rats exhibited a significantly lower Glu/GABA ratio than that of male rats, which may be related to the lower susceptibility of females to the excitotoxic action of Glu *via* sex hormone-potentiated GABA inhibitory neurotransmission. By contrast, this study reported a significant increase in brain GABA in both male and female PPA-treated mice (Table 1 and Fig. 2D) with a remarkable higher Glu/GABA ratio in female treated rats (Fig. 2E), which again could be attributed to the difference in the rodents used. These species differences are extremely relevant in modeling social behavior deficits considering the collaborative and complex nature of human social behavior. Given their intrinsic lack of receptiveness to social interaction, mice may not be the ideal model of ASD, where reductions in social communication and social behavior are core symptoms (Ellenbroek & Youn, 2016; Moy et al., 2008).

LDH is an energy metabolism regulator found in the cytoplasm of many tissues, including the kidney, heart, and brain. Leakage of LDH from the brain to blood is a well-accepted neurotoxic phenomenon. Here, the LDH level increased in PPA-treated female mice but decreased in PPA-treated male mice, which displayed autism-like behavior (Table 1 and Fig. 2F). The significant decrease in brain LDH in PPA-treated male mice (*P* < 0.021) could be used as an index of susceptibility to PPA neurotoxic brain injury. By contrast, the marked but nonsignificant increase in LDH levels in females may act as a protective strategy by increasing the glycolytic influx to compensate for ATP depletion as a marker of impaired energy metabolism (Choi et al., 2018; Ippolito et al., 2017; Ross et al., 2010).

In the brain, CK plays a critical role in maintaining ATP levels in neurons and is considered as a potential biomarker for diagnosing ASD since patients with autism suffer from mitochondrial dysfunction (Khemakhem et al., 2017). A nonsignificant elevation of CK was present in PPA-treated male mice compared with that in the control (Table 1 and Fig. 2G), whereas this declined in PPA-treated female mice. This result was supported by El-Ansary, Bacha & Kotb (2012) and can be explained by an increase in Ca^2+^/Mg^2+^ and Na^+^/K^+^ ATPase activities as well as a decrease in the expression of mitochondrial electron transport chain complexes, which was demonstrated in many brain areas of children with autism compared with that in controls (Chauhan et al., 2011). Al-Mosalem et al. (2009) also noticed increased CK levels as well as reduced ATP levels in the plasma of Saudi patients with autism. This again could be attributed to the absence of ATP depletion in PPA-treated females through the much higher neuroprotective glycolytic influx previously discussed.

Oxidative stress is a condition that develops when ROS, including superoxide, H_2_O_2_, and OH radicals, accumulate in the cells or the antioxidant system fails (Pizzino et al., 2017). The brain is particularly vulnerable to oxidative stress because it contains high levels of lipids and consumes large amounts of energy as well as having limited antioxidant ability (Rossignol & Frye, 2014). GSH is the most powerful nonenzymatic antioxidant and serves a variety of roles (Pizzino et al., 2017), including protecting cells from free radicals, catalysis, metabolism, and transport and playing a role in immunity, cell proliferation, and apoptosis (Lu, 2013). Low GSH levels have been linked to neurological diseases such as Parkinson’s disease, schizophrenia, and autism (James et al., 2006). This study showed a decrease in the level of GSH in both PPA-treated male and female groups compared with that in the control (Table 1 and Fig. 2H), and therefore the GSH depletion in the present study is in good agreement with results by El-Ansary, Bacha & Kotb (2012).

Catalase and GST play a critical part in the antioxidant defense mechanisms by detoxifying xenobiotics and inactivating a wide range of endogenous oxidative stress products (Mandic-Maravic et al., 2019). In recent years, a significant reduction in GST activity has been observed in patients with ASD as compared with typically developed controls (Mandic-Maravic et al., 2021). Here, GST levels in PPA-treated male mice (0.26 ± 0.04 U/ml) were much lower than those in controls (0.31 ± 0.04 U/ml). However, these levels are considerably increased in females, which may assist in the detoxification of the neurotoxic dose of PPA (Table 1 and Fig. 2I). This could be related to females being less susceptible to autism as an acquired cellular detoxification deficiency syndrome (James, 2018). Furthermore, this result could be supported through the work of Das et al. (1981), who reported much higher GST levels in female rats compared with those in males during postnatal development. Recently, Abuaish et al. (2021a) demonstrated that female rats exhibited a significant increase in levels of GST and GSH in response to PPA toxicity compared with those in PPA-treated male rats.

As an enzyme antioxidant system, catalase reduces the buildup of H_2_O_2_ in cells, especially in the brain (Pizzino et al., 2017). Schizophrenia and other illnesses have been related to catalase deficiency or low activity (Góth & Vitai, 1996). Indeed, catalase is known to be effective in preventing neuronal degeneration (Busciglio & Yankner, 1995). The PPA-treated male and female mice in this study showed elevated catalase activity and this could be attributed to the harmful impact of PPA (Table 1 and Fig. 2J). An increase in catalase as a marker of oxidative stress was repeatedly reported in several studies (Kalemci et al., 2017; Niu et al., 2018). Collectively although differences in most of the measured variables make any biologically important difference between the neurochemistry of PPA-treated male and female mice difficult to prove, this study suggests that sex chromosomal gene dosage and sex hormone levels may play a role in determining sex-specific liability thresholds. However, considerably more research is needed to definitively identify the most important players and elucidate the precise mechanisms whereby these sex-specific factors modulate ASD phenotype presentation (Werling & Geschwind, 2013).

The change in the relative abundance of certain fecal bacteria strains in PPA-treated male and female mice compared with that in healthy controls is shown in Table 2. Although the abundance of *Staphylococcus* noticeably increased in both sexes, *Moraxell* a species were completely absent in the male cohort. Moreover, a marked difference was present in the abundance of gut bacteria of control males and females compared with respective PPA-treated groups showing much lower abundance of *Moraxella* species. This is in good agreement with the recent work of Al Dera et al. (2021), which reported a decrease in the abundance of certain bacteria, including *Moraxella*, in individuals with autism and the PPA-rodent model of ASD (Busciglio & Yankner, 1995). Developing children are known to have a higher abundance of *Moraxella* than that in individuals with ASD, who had 31.9% reduced abundance of this bacterial species (Forsyth et al., 2020). In a mouse model of ASD fed a casein and gluten-rich diet, the abundance of fecal *Moraxella* was shown to be reduced concomitantly with a large increase in zonulin, a measure of gut leakiness as comorbidity in ASD (Al Dera et al., 2021). Lower abundance of *Moraxella* in the PPA-rodent model was attributed to the leaky gut as an accepted phenomenon relating gut microbiota to brain disorders *via* the GBA. This, the increasing trend of *Moraxella* abundance in treated female mice, and the complete absence in the male rodent model could help to suggest that female mice as a rodent model of autism had a lower tendency to develop leaky gut as a contributor of autistic features *via* the GBA. Moreover, the higher gram-positive/gram-negative bacterial ratio in females could be easily related to the resistance to developing gut leakiness compared with that in the male rodent model of ASD. Much higher levels of lipopolysaccharide as metabolites of gram-negative bacteria in males can easily pass the intestinal barrier and cause inflammation affecting the brain through altering cytokine levels (Srikantha & Mohajeri, 2019). This could demonstrate the role of sex in the marked susceptibility of males to developing autistic characteristics.

**Table 2:** Changes in bacteria growth in the feces of control and PPA treated male and female mice groups.

**Group**	**Day**	***Staphylococus*** **and/or** ***Bacilli*** **(Gram** ^+^ **cocci/rod or Gram** ^−^ **rod)**	***Enterobacteriacea*** **(Gram** ^−^ **rod, lactose fermenters)**	**Gram**^+^/ Gram^−^**rod and cocci**	***Clostridium botulinum*Gram**^+^, ** rod-shaped**	***Moraxella spp***
**Control Male**	1	–	–	–	–	++++
	2	–	–	–	–	+++++
	3	–	–	–	–	+++++
**PPA- Treated Male**	1	+++	+	–	–	–
	2	++++	+	–	–	–
	3	++	+	–	–	–
**Control Female**	1	++	–	–	–	+++
	2	++	–	–	–	–
	3	+	–	–	–	++
**PPA-Treated Female**	1	++++	++++	++	–	++
	2	+++	–	++	–	+++
	3	+++	+++	++	–	+++

**Table 3:** Alterations in repetitive behavior, cognitive ability, and physical-neural coordination capability in PPA treated male and female mice groups compared to control (*N* = 6).

**Indicators**	**Parameters**	**Groups**	**Mean ± S.D.**	**Significancy**
**A.** **Indicators of repetitive behavior and anxiety**	**Number of Squares crossed**	Control Male	207.47 ± 37.09	
Control Female	167.17 ± 47.06	
PPA Treated Male	265.67 ± 72.50	**
PPA Treated Female	262.33 ± 65.23	*
**Wall rear**	Control Male	19.17 ± 3.69	
	Control Female	17.00 ± 5.29	
	PPA Treated Male	27.00 ± 5.29	**
	PPA Treated Female	27.67 ± 7.64	*
**Rear**	Control Male	6.67 ± 7.02	
	Control Female	6.00 ± 5.57	
	PPA Treated Male	8.00 ± 5.00	*
	PPA Treated Female	8.00 ± 6.56	*
**Locomotion Duration (sec)**	Control Male	214.33 ± 48.69	
	Control Female	193.33 ± 13.43	
	PPA Treated Male	282.00 ± 19.08	**
	PPA Treated Female	266.33 ± 14.29	*
**Immobility Duration (sec)**	Control Male	88.67 ± 43.75	
	Control Female	70.67 ± 13.65	
	PPA Treated Male	24.67 ± 15.04	*
	PPA Treated Female	29.33 ± 16.80	*
**B.** **Indicators of cognitive ability**	**Total latency time (La)**	Control Male	110.17 ± 9.56	
		Control Female	98.67 ± 5.57	
		PPA Treated Male	135.67 ± 5.09	**
		PPA Treated Female	122.50 ± 5.58	**
	**Number of crossings during light and sound stimulus (St)**	Control Male	2.17 ± 0.75	
		Control Female	1.83 ± 0.75	
		PPA Treated Male	0.50 ± 0.55	**
		PPA Treated Female	1.33 ± 0.52	
	**Number of reinforced crossings during the shock (Re)**	Control Male	22.00 ± 1.90	
		Control Female	12.17 ± 1.94	
		PPA Treated Male	21.50 ± 5.58	
		PPA Treated Female	18.67 ± 2.88	**
	**Number of no crossing during the shock (Tr)**	Control Male	2.33 ± 0.52	
		Control Female	2.83 ± 0.75	
		PPA Treated Male	3.17 ± 0.75	*
		PPA Treated Female	3.67 ± 1.03	
**C.** **Indicator of physical and coordination capability**	**Motor voltage measurement by wheels**	Control Male	3.67 ± 1.03	
		Control Female	3.17 ± 0.75	
		PPA Treated Male	2.83 ± 0.75	
		PPA Treated Female	2.33 ± 0.52	*

**Figure 3:**
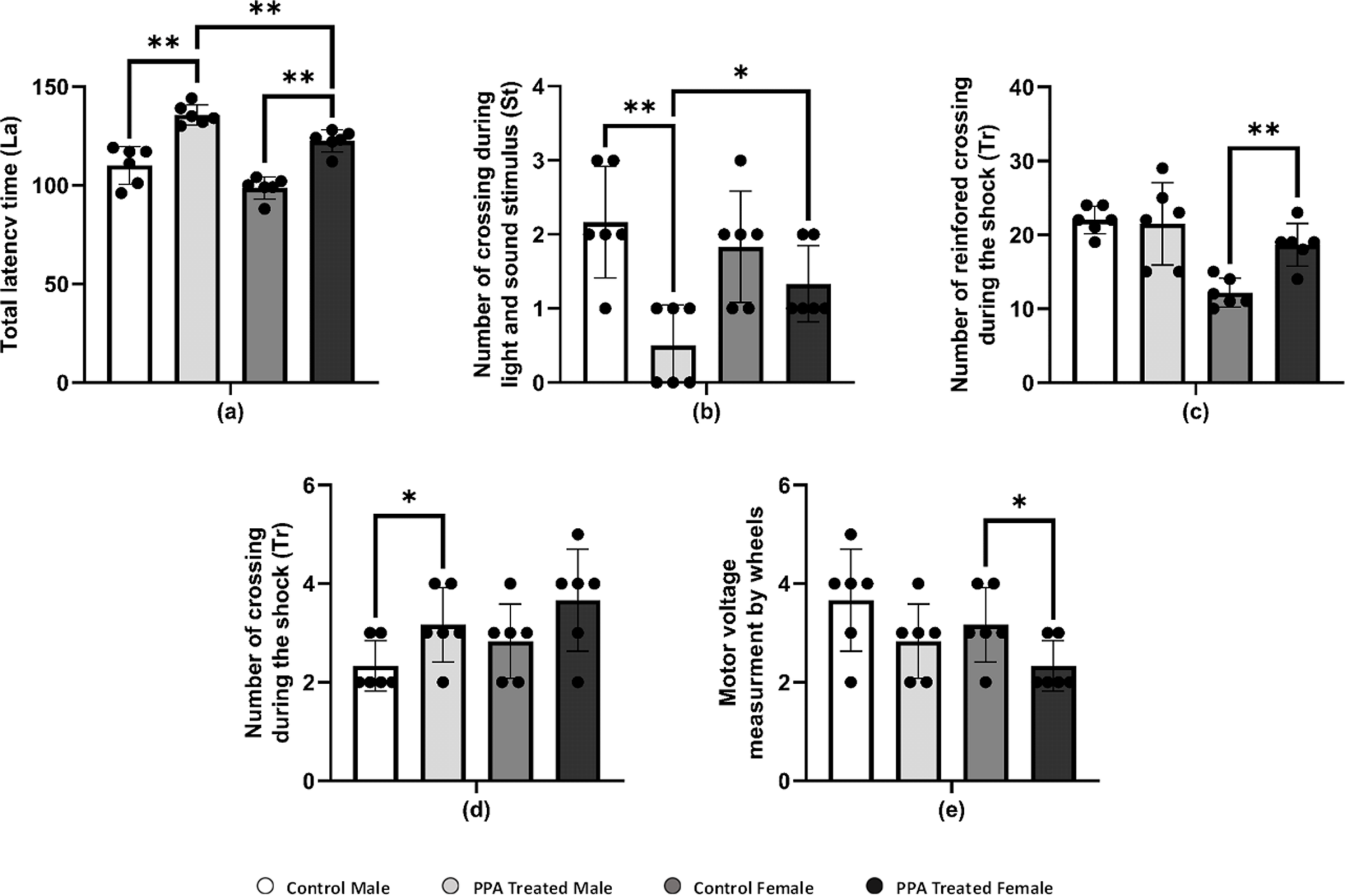
Alterations in repetitive behavior, cognitive ability, and physical–neural coordination capability in PPA treated male and female mice groups compared to control (*N* = 6). One asterisk (*), two asterisks (**), and three asterisks (***) show statistically significant differences at *P* < 0.05, *P* < 0.01 and *P* < 0.001, respectively from the control group by *t*-test.

PPA administration is recognized as being able to impair social behavior and some cognitive tasks and induce convulsions and seizures, oxidative stress, and neuroinflammatory response in treated rodent brains (Shultz et al., 2008; MacFabe, 2012; Shultz et al., 2009). PPA-treated mice significantly crossed a greater number of squares on the locomotion monitor, indicating more horizontal distance was crossed compared with that by control mice (Table 3A). Similar to the horizontal distance result, the number of vertical movements as wall rearing and center raring was also significantly increased in PPA-treated mice relative to controls. In addition, the total locomotion duration was significantly increased in PPA-treated mice, whereas immobility duration was markedly decreased in PPA-treated mice in both genders when compared with that in controls (Table 3A). Thus, the obtained data clearly indicates repetitive behavior and anxiety that represent core autistic-like behaviors in PPA-treated mice, which confirm the efficiency of the ASD mice model used. Similar findings were reported by Rattanapornsompong et al. (2019) and supported by Sagheer et al. (2018), who reported that cerebellar neural bases for both social and motor abnormalities may be shared, and that motor dysfunction may contribute to social and communication deficiencies in ASDs. Similarly, cognitive skills are severely impaired in patients with ASD. Considerable efforts conducted by many studies have increased the understanding of the cognitive deficits in patients with ASD in different cognitive domains, such as the inability to attribute mental states, beliefs, intents, memory, planning, processing speed, and attention. In the current study, PPA-treated mice showed significantly more latency time for both genders compared with each corresponding control group, indicating the defect in learning and memorizing and thereby exposing the PPA-treated mice to harm (Table 3B and Fig. 3A). During light and sound stimulus both sexes of PPA-treated mice showed a smaller number of crossings compared with that in the control where they could comprehend the stimulus and run to avoid the shock; the decrease in crossings of PPA-treated male mice was significant, indicating a stronger cognitive defect (Table 3B and Fig. 3B). Similarly, PPA-treated female mice showed a significant increase in the number of reinforced crossings during the shock compared with that in the control, making them faster learners than PPA-treated male mice (Table 3B and Fig. 3C). The number of no crossings during the shock was significantly higher in PPA-treated male mice compared with that in the control, making males more susceptible to be harmed by shocks then females were because of the higher degree of cognitive defects and decreased learning and memorizing in PPA-treated male mice (Table 3B and Fig. 3D). These findings are supported by a systematic review by Velikonja, Fett & Velthorst (2019).

Moreover, there is an increasing belief that children with ASD exhibit motor impairments such as poor balance and postural control, incoordination, and unsteady gait (Kaur & Bhat, 2019). In this context, we performed a rotarod test in a mice model of ASD to investigate rodent motor coordination, which was particularly sensitive in detecting cerebellar dysfunction (Shiotsuki et al., 2010). PPA-treated male and female mice both exhibited a significant decrease in the duration of immobility, indicating motor impairments when compared with that in the control (Table 3C and Fig. 3E).

## Conclusions

The biochemical findings of this study partially explain why male rats are more likely to develop biochemical and behavioral autistic features in response to a neurotoxic dose of PPA, although differences in the majority of the analyzed biomarkers make biologically relevant variation in the neurochemistry of PPA-treated male and female mice difficult to confirm. More research is required to definitively identify the most important contributors and the precise processes whereby these sex-specific factors influence ASD clinical presentation.

##  Supplemental Information

10.7717/peerj.15488/supp-1 Supplemental Information 1 Alterations in repetitive behavior in PPA treated male and female mice groups compared to control (*N* = 6) * and ** show statistically significant difference at *P* < 0.05 and *P* < 0.01, respectively from the control group by *t*-test.

10.7717/peerj.15488/supp-2 Supplemental Information 2 Raw data

10.7717/peerj.15488/supp-3 Supplemental Information 3 Author Checklist

## Additional Information and Declarations

### Data Availability

The following information was supplied regarding data availability: The raw measurements are available in the Supplemental Files.
