# Supplementary material for: Unveiling sex-based differences in developing propionic acid-induced features in mice as a rodent model of ASD
Source: PeerJ. 2023 Jun 13;11:e15488. doi: 10.7717/peerj.15488 (PMC10274690; doi:10.7717/peerj.15488)

Figure: Alterations in repetitive behavior in PPA treated male and female mice groups compared to control (N=6). * and ** show statistically significant difference at P < 0.05 and P < 0.01, respectively from the control group by t-test.

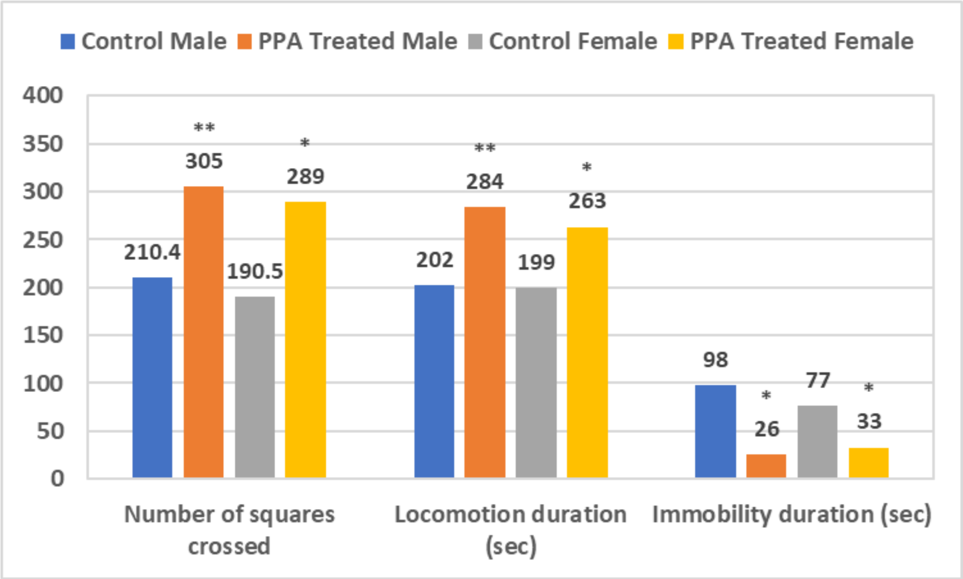

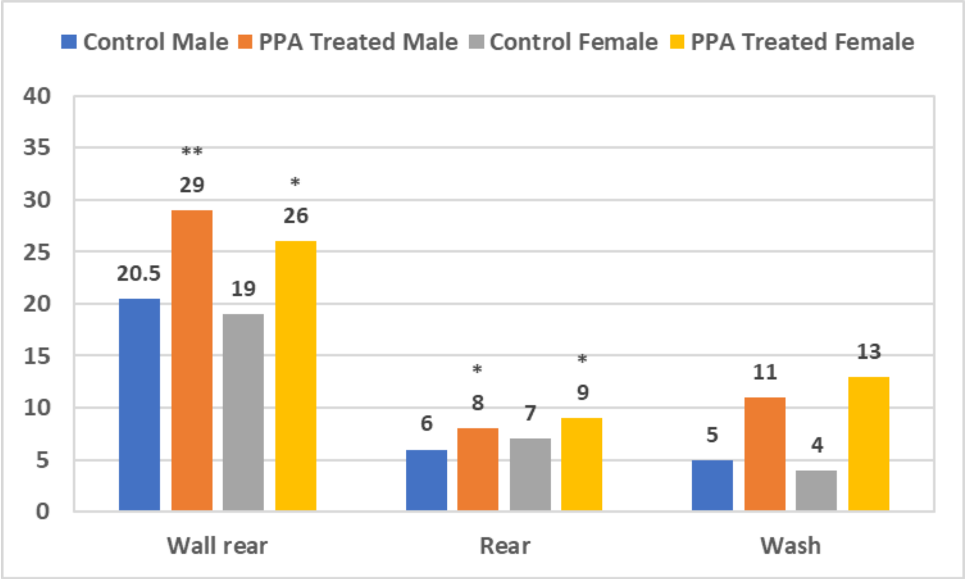

Supplement: Supplemental Information 1 — * and ** show statistically significant difference at P < 0.05 and P < 0.01, respectively from the control group by t-test. [file peerj-11-15488-s001.pdf]
